# TBX2 Drives Neuroendocrine Prostate Cancer through Exosome-Mediated Repression of miR-200c-3p

**DOI:** 10.3390/cancers13195020

**Published:** 2021-10-07

**Authors:** Girijesh Kumar Patel, Sayanika Dutta, Mosharaf Mahmud Syed, Sabarish Ramachandran, Monica Sharma, Venkatesh Rajamanickam, Vadivel Ganapathy, David J. DeGraff, Kevin Pruitt, Manisha Tripathi, Srinivas Nandana

**Affiliations:** 1 Department of Cell Biology and Biochemistry, Texas Tech University Health Sciences Center, Lubbock, TX 79430, USA; girijesh.patel@ttuhsc.edu (G.K.P.); sayanika.dutta@ttuhsc.edu (S.D.); mosharaf.mahmud-syed@ttuhsc.edu (M.M.S.); s.ramachandran@ttuhsc.edu (S.R.); vadivel.ganapathy@ttuhsc.edu (V.G.); 2 Department of Immunology and Molecular Microbiology, Texas Tech University Health Sciences Center, Lubbock, TX 79430, USA; monica.sharma@ttuhsc.edu (M.S.); kevin.pruitt@ttuhsc.edu (K.P.); 3 Earle A. Chiles Research Institute, Providence Cancer Institute, Portland, OR 97213, USA; venkatesh.rajamanickam@providence.org; 4 Department of Pathology and Laboratory Medicine, Pennsylvania State University College of Medicine, Hershey, PA 17033, USA; ddgraff@pennstatehealth.psu.edu; 5 Department of Urology, Texas Tech University Health Sciences Center, Lubbock, TX 79430, USA

**Keywords:** exosomes, N-MYC, SOX2, TBX2, treatment-induced neuroendocrine prostate cancer, miR-200c-3p

## Abstract

**Simple Summary:**

An estimated ~25–30% of patients with advanced prostate cancer (PCa) develop the aggressive and lethal form of the disease known as treatment-induced neuroendocrine prostate cancer (t-NEPC). Owing to lack of treatment options, the identification of the underlying molecular mechanisms that propagate the t-NEPC phenotype is critical towards developing novel therapeutic strategies against advanced PCa. Further, the roles of extracellular vesicles (exosomes) and microRNAs—an increasingly recognized and key mode of propagation of the NEPC phenotype—remain elusive. Our studies reveal that TBX2 promotes SOX2- and N-MYC- driven t-NEPC through regulation of the intermediary factor—miR-200c-3p; and that TBX2/miR-200c-3p/SOX2/MYCN signaling can promote t-NEPC via both intracellular and exosome-mediated intercellular mechanisms.

**Abstract:**

Deciphering the mechanisms that drive transdifferentiation to neuroendocrine prostate cancer (NEPC) is crucial to identifying novel therapeutic strategies against this lethal and aggressive subtype of advanced prostate cancer (PCa). Further, the role played by exosomal microRNAs (miRs) in mediating signaling mechanisms that propagate the NEPC phenotype remains largely elusive. The unbiased differential miR expression profiling of human PCa cells genetically modulated for TBX2 expression led to the identification of miR-200c-3p. Our findings have unraveled the TBX2/miR-200c-3p/SOX2/N-MYC signaling axis in NEPC transdifferentiation. Mechanistically, we found that: (1) TBX2 binds to the promoter and represses the expression of miR-200c-3p, a miR reported to be lost in castrate resistant prostate cancer (CRPC), and (2) the repression of miR-200c-3p results in the increased expression of its targets SOX2 and N-MYC. In addition, the rescue of mir-200c-3p in the context of TBX2 blockade revealed that miR-200c-3p is the critical intermediary effector in TBX2 regulation of SOX2 and N-MYC. Further, our studies show that in addition to the intracellular mode, TBX2/miR-200c-3p/SOX2/N-MYC signaling can promote NEPC transdifferentiation via exosome-mediated intercellular mechanism, an increasingly recognized and key mode of propagation of the NEPC phenotype.

## 1. Introduction

Androgen deprivation therapy (ADT) remains the cornerstone of treatment for advanced prostate cancer (PCa). However, relapse occurs invariably in all advanced patients with adenocarcinoma when the disease manifests as castrate-resistant prostate cancer (CRPC). Up to 25% of CRPC then progresses to neuroendocrine prostate cancer (NEPC), characterized by aberrant expression of neuroendocrine lineage markers and loss of androgen receptor signaling [1,2] This phenomenon has interchangeably been referred to in the literature as plasticity, tumor subtype switching, NEPC transdifferentiation, or treatment-induced NEPC (t-NEPC). Although NEPC can arise de novo in rare cases (0.5–2%), t-NEPC is increasingly being recognized as a variant of ADT-resistant CRPC that arises as a consequence of therapy [3,4,5].

These NEPC cells, which occur as scattered foci within the larger backdrop of CRPC, share molecular features with small-cell carcinoma, are aggressive in nature, and constitute a major therapeutic challenge in the clinical management of advanced PCa due to their unresponsiveness to even second-generation ADTs such as enzalutamide. The inherent pathophysiology of the NEPC that exists as scattered foci may therefore offer clues into the development and sustenance of the phenotype. Furthermore, NEPC variants have increased following the use of the newer ADTs such as enzalutamide, compounding the problem, and presenting a clinical conundrum [3,4,5]. Therefore, identifying the relevant molecular/signaling mechanisms that potentiate t-NEPC/NEPC transdifferentiation, and more importantly, deciphering how PCa cells communicate with each other in propagating the NEPC phenotype is central to efforts in designing novel and effective therapeutic modalities against advanced PCa.

Although progress has been made in the recent years in identifying a few molecular drivers of t-NEPC/NEPC transdifferentiation including SOX2 and N-MYC [6,7,8,9,10,11,12,13], the signaling mechanisms that foster the NEPC pathophysiology particularly in the context of the intercellular communication between PCa cells in sustaining the phenotype remains largely elusive.

Extracellular vesicles (EVs), particularly exosomes, have in recent years been recognized as key mediators of cancer progression and metastasis [14,15,16,17]. MicroRNAs (miRs)—a component of the exosomal cargo—are a class of small noncoding RNAs that bind to the target mRNAs, repress gene expression, and play fundamental roles in the development, differentiation, and manifestation of pathophysiological conditions [18,19]. Numerous studies have shown that the transfer of miRs between cells via exosomes is a vital means of communication in cancer [20,21]. However, the roles played by exosomes and miRs in the development of advanced PCa, particularly NEPC transdifferentiation, remain largely unknown [14,15,16]. miR-200c-3p, a miR that has a documented role in cancer progression, is known to negatively regulate epithelial-to-mesenchymal transition (EMT) and metastasis in several malignancies [22,23,24] and is implicated in neuronal pathways [25].

We previously reported that TBX2, a T-box transcription factor, plays a key role in PCa including metastatic progression [26]. In the present study, using in vitro and in vivo models, we report that TBX2 drives SOX2- and N-MYC- mediated NEPC transdifferentiation via the repression of miR-200c-3p and that miR-200c-3p as a mediator is sufficient for TBX2/SOX2/N-MYC signaling to promote NEPC transdifferentiation. Further, our results demonstrate the intercellular exosome-mediated paracrine (non cell-autonomous) mode as a mechanism of NEPC transdifferentiation is supported by TBX2/miR-200c-3p/SOX2/N-MYC signaling in addition to mediating the intracellular (cell-autonomous) changes in neuroendocrine gene expression. In summary, our study reveals a crucial signaling axis downstream of TBX2 that drives the NEPC pathophysiology including exosome-mediated transfer, and our findings could provide critical clues in understanding the molecular/signaling events that drive and propagate therapy resistance in this lethal subset of advanced PCa.

## 2. Materials and Methods

### 2.1. Cell Culture and Treatments

Human PCa cell lines (PC3, C4-2B, LNCaP, and 22Rv1) were maintained in RPMI-1660 and/or DMEM media supplemented with 5–10% fetal bovine serum (FBS) and 1% penicillin and streptomycin at 37 °C in a humidified CO2 (5%) incubator. PCa cells were received from Dr. Leland W. K. Chung, Uro-Oncology Research Program, Department of Medicine, Cedars-Sinai Medical Center, Los Angeles, California, USA. Viral packaging cell line (293FT) was a generous gift from Dr. Vadivel Ganapathy, Department of Cell Biology and Biochemistry, Texas Tech University Health Sciences Center, Lubbock. Cell lines used in the study were intermittently evaluated in-house and were free of mycoplasma contamination.

### 2.2. Exosome Isolation and Characterization

For exosomes isolation, PCa cells (PC3^Neo^/PC3^TBX2DN^, C4-2B^Neo^/C4-2B^TBX2DN^, and LNCaP^Neo^ and LNCaP^TBX2^) were grown in regular media. At 70% confluency, fresh media supplemented with 5% exosome-depleted FBS (Gibco, Grand Island, NY, USA) were replaced. After 36 h, conditioned media were collected to fractionate the EV (e.g., apoptotic bodies (ABs), microvesicles (MVs), exosomes) and soluble factors (SFs) as described earlier [27]. The EVs were washed with PBS, and protein-based quantification was performed using protein DC assay kit (Bio-Rad, Hercules, CA, USA). Size distribution analysis of all EVs was performed using Zetasizer ZSP (Malvern Panalytical, Malvern, UK) at 25 °C. Multiple scans were acquired from each preparation and averaged. Data are represented from an average of three biological replicates. The size of exosomes was also determined using transmission electron microscope (TEM). In brief, 5 µL of the exosome sample was placed onto a copper grid (200-mesh) with carbon-coated formvar film (Ted Pella, Redding, CA, USA) and incubated for 2 min at room temperature (RT). After removal of excess liquid using blotting paper, 5 μL (2% *w/v*) of uranyl acetate solution (Electron Microscopy Sciences, Hatfield, PA, USA) was added for negative staining for 1 min at RT. The grid was washed 2 times with 5 μL of filtered molecular grade water, air-dried, and stored. Images were acquired using Hitachi H-7650 transmission electron microscope at 60.0 KV and 20,000× magnification at Texas Tech University College of Arts and Sciences Microscopy facilities.

### 2.3. RNA Isolation, cDNA Synthesis, and quantitative real-time RT-PCR (qRT-PCR) Analysis

RNA was isolated using the RNeasy Mini Kit (Qiagen Inc., Valencia, CA, USA). After DNA digestion and quantified using NanoDrop One (Thermo Fisher Scientific, Waltham, MA, USA), a total of 2 μg RNA was transcribed into cDNA using the high-capacity RNA-to-cDNA kit (Applied Biosystems, Carlsbad, CA, USA). Quantitative real-time RT-PCR (qRT-PCR) was performed in a QuantStudio 12K Flex real-time PCR system using powerUP SYBR Green reagent (Applied Biosystems) with a specific set of primers listed in Table S1. The relative amount of mRNA expression was normalized with β-actin. For the microRNA (miR) assay, total miR was isolated using mirPremier miR isolation kit (Sigma-Aldrich, St. Louis, MO, USA), and cDNA was synthesized using miR-first-strand synthesis kit (Agilent Technologies, Santa Clara, CA, USA). The small nuclear RNA (U6) was used to normalize the miRs expression. List of miR primers is given in the Table S2.

### 2.4. Protein Extraction and Quantification

Protein extracts from PCa cells and exosomes were prepared using radio-immunoprecipitation assay (RIPA) buffer in the presence of protease and phosphatase inhibitors cocktails (Pierce, Rockford, IL, USA) after brief sonication. Protein concentration was measured using the protein DC assay kit (Bio-Rad) in a 96-well plate. Absorbance was recorded at 750 nm using an iMark plate reader (Bio-Rad), and protein concentration was calculated using BSA as a standard.

### 2.5. SDS-PAGE and Western Blot Analysis

Equal amounts of protein (ranging from 20 to 100 μg) were resolved onto 10% SDS-PAGE gel and electroblotted onto PVDF membrane. Membranes were blocked with 5% BSA in Tris buffer saline supplemented with 0.1% Tween (TBST) for 1 h at RT and probed with different primary antibodies including anti-CD81 (sc-166029) and anti-SOX2 (sc-365823) from Santa Cruz Biotechnology, Inc. (Dallas, TX, USA), anti-CD9 (Cell Signaling Technology, Danvers, MA, USA, CST#13403) for overnight at 4 °C. N-MYC antibody was kindly provided by Dr. Min Kang, PharmD, TTUHSC. After washing, membranes were probed with horseradish peroxidase-conjugated secondary antibodies (rabbit/mouse, CST, Danvers, MA, USA) at RT for 1 h, and signals were detected using west pico-chemiluminescent kit (Thermo Fisher Scientific) under ChemiDoc Touch Imaging System (Bio-Rad, Hercules, CA, USA). β-actin was used as a loading control for the protein.

### 2.6. In Silico Analysis for microRNA Putative Targets

To predict the microRNA targets, we used multiple in silico analysis tools including miRDB [28] and targetscan [29]. Based on the miR’s abundance and higher targeting score for those genes which were consistently altered in TBX2-modulated PCa cells, miR-200c-3p was considered for the further study. In addition, miRNET2.0 [30] was utilized to create a network graph showing the genes interaction of the top 5 overexpressed and top 5 underexpressed miRs that were identified following next-generation sequencing of exosomes from PC3^TBX2DN^ and PC3^Neo^ cells. We used degree filter 2.0 and shortest distance filter 2.0 across all nodes to remove nodes with low degree centrality and to extract minimally covered subgraphs [30].

### 2.7. Plasmid Propagation, Transduction, and Modulation of miR-200c-3p Expression

The custom constructs for miR-200c-3p overexpression (Cat# mh10263), inhibition (miROff-200c-3p, Cat #mh30308), and their respective controls (Cat# m001 and Cat#m007) were purchased from Applied Biological Material Inc. (Richmond, BC, Canada). These lentiviral miR-constructs along with the packaging plasmid (pCMVdeltaR8.2dvpr, addgene#8455) and envelop plasmid (pCMV-VSV-G, addgene#8454) were used for lipofectamine 2000-mediated transfection of 293FT cells to produce lentiviral pseudotyped particles. The supernatant containing viral particles was collected after 36 h of transfection, centrifuged 300× *g* for 5 min, and filtered through a 0.45 μm membrane and used to infect the target cells (TBX2-modulated PCa cells) in presence of 10 μg/mL of hexadimethrine bromide (polybrene, Sigma-Aldrich, St. Louis, MO, USA). Infection with viral particles was repeated three times, and selection was performed using 500 μg/mL G418 (neomycin) for 2 weeks and completed once negative control cells showed 100% death.

### 2.8. Chromatin Immunoprecipitation (ChIP) Assays

Chromatin immunoprecipitation (ChIP) assays were performed as described previously [31] with slight modifications. The PC3^Neo^ and PC3^TBX2DN^ cells were grown to about 80% confluency in 150 mm dishes. For DNA-protein crosslinking, formaldehyde was used directly in the culture media at a final concentration of 1% for 8 min at RT and quenched by addition of glycine at a final concentration of 0.125 M for 5 min at RT. Thereafter, media were removed, and cells were washed 3 times with cold PBS. Cells were harvested and washed twice with PBS containing protease inhibitor cocktail (Pierce, Rockford, lL, USA). The cell pellets were lysed using SDS-lysis buffer (50 mM Tris-HCl pH 8.0, 10 mM 0.5 M EDTA, and 1% SDS) supplemented with 1× protease inhibitor cocktail and sonicated using a Bioruptor (Diagenode Inc., Denville, NJ, USA). The soluble chromatin fraction was quantified, and 100 μg of chromatin was incubated for 2 h at 4 °C with anti-TBX2 and IgG antibody. Thereafter, prewashed Dynabeads protein G (11 μL) (Invitrogen #1003D, Carlsbad, CA, USA) was added to the chromatin-antibody mixture and incubated at 4 °C on a rotator for 2 h. ChIP mixture was washed five times with low salt buffer (20 mM Tris-HCl, pH 8.1; 0.1% SDS, 1% Triton X-100, 2 mM EDTA, and 150 mM NaCl), four times with high salt buffer (20 mM Tris HCl pH 8.1, 0.1% SDS, 1% Triton X-100, 2 mM EDTA, and 500 mM NaCl), and finally washed with TE buffer (10 mM Tris HCl pH 8 and 1 mM EDTA). Reverse crosslinking was performed overnight at 65 °C, followed by RNase A (Sigma-Aldrich) at 37 °C for 2 h, and proteinase K digestion (Sigma-Aldrich) at 55 °C for 2 h. DNA was purified using the PCR purification kit (Qiagen, Hilden, Germany) and amplified by end-point PCR and qRT-PCR using specific primers pairs listed in Table S2.

### 2.9. Tumor Xenograft Experiments in Mice

All animal experiments were performed as per the protocol approved by the Institutional Animal Care and Use Committee (IACUC). Luciferase-tagged PCa cells (PC3^Neo^ or PC3^TBX2DN^) were engrafted (1 × 10^6^ per mouse) in the anterior prostate lobes of 4–6 week-old NUDE mice. Xenograft tumors were resected after 10 weeks, and tumor size and volume were recorded [26]. Volume measurement was performed using the formula

(*X* × *Y*2)/2, (1)

where *X* is the larger and *Y* is the smaller of the two dimensions. All the tissues were fixed in 10% neutral-buffered formalin for 6 h at RT and embedded in paraffin for further study.

### 2.10. Immunohistochemistry of Tumor Tissues

To determine the expression pattern of the neuroendocrine markers in PC3^TBX2DN^ and PC3^Neo^ xenograft tissues, slides were stained with anti-SOX2 (CST#14962) and anti-N-MYC (CST#51705) antibodies (Cell Signaling Technology, MA, USA). The list of all the antibodies used in this study is provided in Table S3. In brief, the slides were deparaffinized by incubation in xylene two times (10 min each). Thereafter, slide sections were hydrated by sequential incubation in decreasing concentration of ethanol (100%–10%) 5 min in each and rinsed with running water. Antigen unmasking was performed in decloaking chamber using 1× antigen unmasking buffer (H-3300, Vector Lab, Burlingame, CA, USA). Thereafter, blocking of the endogenous peroxidase was performed for 10 min with Bloxall (SP-6000, Vector Lab). To block the tissue sections, normal goat or horse serum (Vector Lab) diluted in PBS (3 drops in 10 mL) were used for 40 min. Slides were incubated with the primary antibodies against SOX2 and N-MYC in a humidified chamber for overnight at 4 °C. Thereafter, slides were washed with PBS and incubated for 45 min with biotinylated antibody stock (1 drop) and 3 drops of stock serum in 10 mL PBS. After washing with TBST for 5 min, premixed Vectastain ABC (2 drops reagent A in 5 mL PBS and 2 drops of reagent B) were added onto the slides and incubated for 30 min. Then, slides were washed with TBST and PBS (5 min with each). To develop the desired color, peroxidase substrate was added, and slides were washed with water. Hematoxylin (H-3401, Vector Lab) counterstain was added and incubated for 1 min at RT and washed with water, and then slides were dipped 5 times in 1× Techa’s bluing solution and washed with water. Slides were mounted with Vectamount (H-5000, Vector Lab), and coverslip was applied after dehydration by incubating in 100% ethanol 2 times (10 s each) and then in xylene two times (10 s each) and air-dried. For negative control immunostaining, tissue sections were probed with normal mouse IgG in parallel (Santa Cruz Biotechnology, Dallas, TX, USA). Fiji, an image-processing package (https://imagej.net/software/fiji/, version 2.3.0, accessed on 10 September 2021), was used to analyze the optical density of the stained slides using the formula

OD = log (Max intensity/Mean intensity),(2)

where max intensity = 255 for 8-bit images.

### 2.11. Exosome Internalization and Fluorescence Microscopy

22Rv1 cells were grown on 12 mm glass coverslips placed into a 24-well plate. After attachment, cells were starved overnight. The PKH67 labeled (green) exosomes (20 μg/mL) from TBX2 modulated PCa cells were incubated for 8 h with starved cells. Thereafter, cells were washed with PBS (3 times) and fixed with 4% paraformaldehyde for 10 min at RT, followed by 3 washes with PBS. The glass coverslips containing the fixed cells were mounted on glass slide using ProLong gold-antifade containing DAPI (Invitrogen, Eugene, OR, USA). Z-stack TD images were acquired using Nikon A1 R confocal microscope at the Imaging Core Facility of Texas Tech University Health Sciences Center, Lubbock, TX, USA.

### 2.12. Statistical Analysis

All the data represented were obtained from three biological replicates and expressed as mean ± SD. Wherever appropriate, PRISM GraphPad 9.1 (San Diego, CA, USA) was used to perform all the statistical analysis. For the two group comparisons, data were subjected to unpaired two-tailed Student’s *t*-tests, while one-way ANOVA was used for the comparison among more than two groups. The *p* values ≤ 0.05 was considered to be statistically significant. In order to determine the potential association between TBX2, MYCN, and SOX2 in human PCa samples obtained from c-bioportal [32,33], Spearman and Pearson correlation coefficients were analyzed along with the respective *p* values.

## 3. Results

### 3.1. TBX2 Regulates Expression of NEPC Markers in PCa via Cell-Autonomous and Exosome-Mediated Non Cell-Autonomous Mechanisms

**Figure 1:**
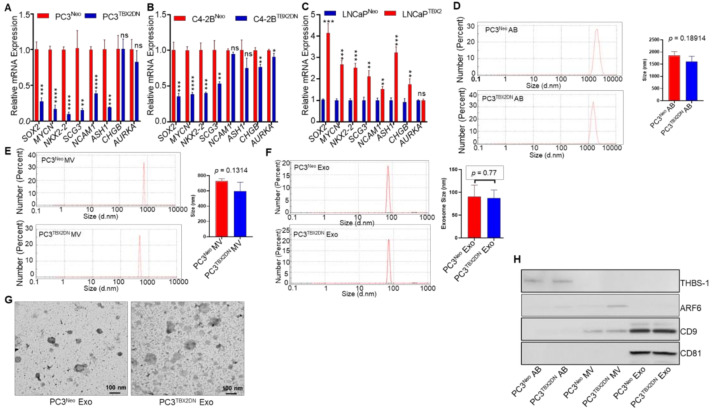

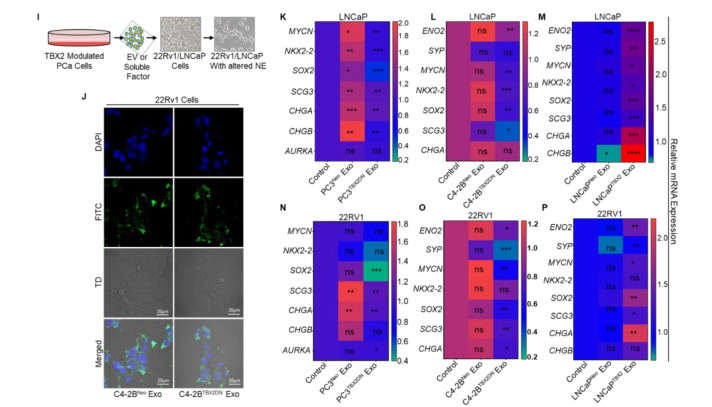
TBX2 regulates expression of neuroendocrine markers in PCa via cell-autonomous and exosome-mediated non cell-autonomous modes: (**A–C**) Graph summarizing the quantitative real-time RT-PCR (qRT-PCR) results comparing the expression of neuroendocrine markers in TBX2-modulated PCa cells: (**A**) PC3^Neo^ and PC3^TBX2DN^; (**B**) C4-2B^Neo^ and C4-2B^TBX2DN^; and (**C**) LNCaP^Neo^ and LNCaP^TBX2^ overexpressing cells. Data represent the average of triplicate values ± S.D.; Student’s unpaired 2-tailed *t*-tests were performed to compare the two groups. *, *p* ≤ 0.05 **, *p* < 0.01; ***, *p* < 0.001, and ****, *p* < 0.0001, and ns indicates not significant. Extracellular vesicles were isolated from PC3^Neo^ and PC3^TBX2DN^ cells and characterized according to: (**D**) size of apoptotic bodies (ABs); (**E**) microvesicles (MVs); (**F**) exosomes (Exo); diameter shown in nanometers (d.nm) (**G**) TEM image (20000×) of exosomes. Bars represents 100 nm size; (**H**) purity of extracellular vesicle (EV) preparation was assessed using Western blot assay probed with Thrombospondin 1 (THBS1) for ABs, ADP-ribosylation factor 6 (ARF6) for MVs, and cluster of differentiation 9 (CD9) protein and cluster of differentiation 81 (CD81) protein as exosome-specific markers; (**I**) schematic representation of the treatment strategies with EVs or soluble factors (SFs); (**J**) Z-stack transmitted detector (TD) images (60×) from the confocal microscope indicating that the majority of exosomes stained with PKH67 (green) are internalized and are present in the cytoplasm of the recipient (22Rv1) cells. Some of the exosomes (green) can also be seen near the nuclei (blue) stained with 4’,6-diamidino-2-phenylindole (DAPI). Bars represents 20 nm size; (**K–P**) heatmap summarizing the results from qRT-PCR analysis comparing the expression of neuroendocrine (NE) markers in LNCaP or 22Rv1 cells following treatment with exosomes (20 μg/mL) derived from TBX2-modulated PCa cells (added a total of two times on alternate days) or with vehicle control. Data represent the average of triplicates values ± S.D.; Student’s unpaired 2-tailed *t*-tests were performed to compare the two groups or one-way ANOVA for more than two groups. *, *p* ≤ 0.05 **, *p* < 0.01; ***, *p* < 0.001 ****, and *p* < 0.0001, and ns indicates not significant. The uncropped Western blot images can be found in Figure S7.

We previously reported that TBX2 is upregulated in human PCa, and that the progression of human PCa xenografts to CRPC is associated with increased TBX2 expression [26]. A recent bioinformatics-based analysis of publicly available human NEPC datasets identified TBX2 as a key upstream regulator of several upregulated genes in human NEPC [34]. Accordingly, we endeavored to determine the impact of genetic modulation of TBX2 on the dysregulation of markers associated with the development of NEPC. Relative to respective Neo controls, PC3^TBX2DN^ and C4-2B^TBX2DN^ cells exhibited significantly reduced expression of neuroendocrine markers (Figure 1A,B), while LNCaP^TBX2^ cells exhibited increased expression of neuroendocrine markers (Figure 1C). Specifically, TBX2 modulation—by the Dominant Negative (DN) and overexpression approaches—resulted in the modulation of mRNAs encoding several neuroendocrine markers including *SOX2, MYCN, NKX2-2, SCG3, NCAM1, ASH1, CHGB*, and *AURKA*. Among these markers, we observed that *SOX2*, *MYCN*, *NKX2-2*, and *SCG3* were consistently altered with *TBX2* genetic modulation (by DN and overexpression approaches) across all three human PCa cell lines used, i.e., PC3, C4-2B, and LNCaP (Figure 1A–C). These results suggested that TBX2 in PCa cells exerts its effects on NEPC transdifferentiation through intracellular gene expression changes.

Scattered foci of NEPC are often detected within the setting of CRPC [3,5]. It has been reported that in addition to transdifferentiating to NEPC, NEPC cells in turn, can potentiate transdifferentiation of adjacent CRPC cells to NEPC [14,15,16]. Therefore, we reasoned that in addition to orchestrating intracellular changes promoting neuroendocrine transdifferentiation, TBX2 expression may also mediate the non cell-autonomous (intercellular) communication via paracrine effects to promote NEPC transdifferentiation. To test this hypothesis, we isolated EV fractions including apoptotic bodies (ABs), microvesicles (MVs), exosomes, and soluble factors (SFs) from the conditioned media of PC3^TBX2DN^, C4-2B^TBX2DN^, or the respective Neo control cells. Isolated EV fractions from the culture supernatants of PC3^TBX2DN^ or PC3^Neo^ cells were first characterized with regard to size using Zetasizer. We found no significant differences in ABs (1890 vs. 1625 nm), MVs (780 vs. 595 nm) and exosomes (91 vs. 84 nm) isolated from PC3^TBX2DN^ or PC3^Neo^ cell (Figure 1D–F, respectively). In addition, transmission electron microscopy further confirmed that the exosomes from PC3^TBX2DN^ or PC3^Neo^ cells conformed to the established exosomal size range (30–150 nm) and that there were no significant differences in the size (Figure 1G). Western blot analysis of isolated EVs using previously reported markers of ABs (THBS1), MVs (ARF6), and exosomes (CD9 and CD81) [35,36] further confirmed the successful EV fractionation (Figure 1H).

To investigate the potential impact of individual EV fractions and soluble factors (SFs) derived from TBX2 modulated cells on neuroendocrine transdifferentiation, 22Rv1 and LNCaP human PCa cells were treated as depicted in Figure 1I. The efficient internalization of exosomes was confirmed by PKH67 staining and confocal microscopy (Figure 1J). Treatment of 22Rv1 and LNCaP human PCa cells with larger vesicles (e.g., ABs or MVs) and soluble factors (SFs) isolated from PC3^TBX2DN^, C4-2B^TBX2DN^, or the respective Neo control cells had no significant impact on neuroendocrine marker expression (Figures S1 and S2). However, treatment of LNCaP or 22Rv1 cells with exosomal fractions isolated from TBX2 modulated cells (PC3^TBX2DN^, C4-2B^TBX2DN^, or LNCaP^TBX2^ cells) resulted in significant changes in neuroendocrine marker expression when compared with exosomal fractions isolated from the respective Neo control cells (Figure 1K–P). In agreement with TBX2-mediated intracellular gene expression changes associated with neuroendocrine transdifferentiation, gene expression changes following TBX2DN exosome treatment consistently showed significant decreases in the expression of *SOX2*, *MYCN*, and *NKX2-2* when compared with the respective controls, while conversely, exosomes from LNCaP^TBX2^ consistently enhanced the expression of *SOX2*, *MYCN*, and *NKX2-2*, when compared with the respective controls. Taken together, these results suggest that in addition to the role of TBX2 in promoting transdifferentiation to NEPC in a cell-autonomous manner, TBX2 also drives NEPC transdifferentiation via exosome-mediated communication in an intercellular manner.

### 3.2. miR-200c-3p Is Downstream of TBX2 Signaling in PCa

**Figure 2:**
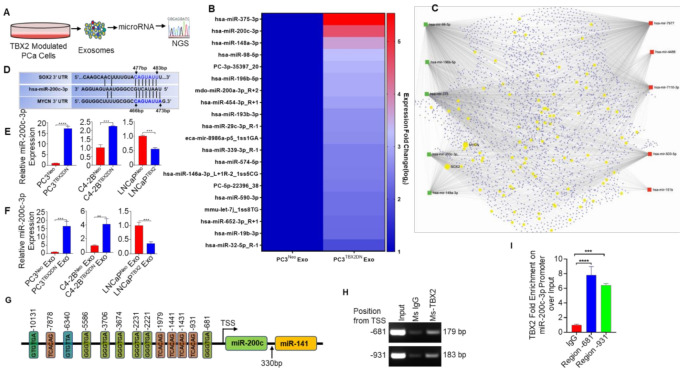
miR-200c-3p is downstream of TBX2 signaling in PCa: (**A**) schematic representing exosome isolation and next generation sequencing (NGS) of exosomal microRNA; (**B**) heat-map depicting the top 20 upregulated miRs in the exosomes derived from PC3^TBX2DN^ cells when compared with PC3^Neo^ cells and normalized log_2_-fold changes are represented; (**C**) miRNET2.0-based analysis [30] showing the interactions amongst the top 5 upregulated miRs (as green squares) and the top 5 downregulated miRs (as red squares) in the exosomes from PC3^TBX2DN^ cells compared with PC3^Neo^ cells. The circular nodes in yellow represent the genes that are enriched in neuronal pathways as found in KEGG and reactome databases. The circular nodes in steel blue represent all other target genes for these differentially expressed miRs. Magnified image is provided in Figure S3; (**D**) in silico analysis showing the 3′ UTRs of SOX2 and MYCN that contain the miR-200c-3p putative target sites (blue); (**E**,**F**) validation of miR-200c-3p using quantitative real-time RT-PCR (qRT-PCR) analysis in: (**E**) TBX2 modulated human PCa cells and (**F**) exosomes derived from TBX2 modulated human PCa cells. Data represent the average of triplicates ± S.D.; Student’s unpaired 2-tailed *t*-tests were performed to compare the two groups **, *p* ≤ 0.01; ***, *p* ≤ 0.001; and ****, *p* ≤ 0.0001; (**G**) in silico analysis showing several TBX2 binding sites on the promoter of miR-200c-3p; (**H**) Chromatin immunoprecipitation (ChIP) assay revealed two TBX2 binding sites located upstream (−681 and −931) of the transcriptional start site (TSS) of miR-200c-3p; (**I**) TBX2 occupancy on miR-200c-3p promoter (located upstream at −681 and −931) analyzed using qRT-PCR and normalized to input. One-way ANOVA was performed where (*n* = 3), ***, *p* < 0.001; ****, *p* < 0.0001.

In addition to being regulators of intracellular gene expression, miRs are recognized as critical mediators of gene expression in adjacent cell populations following exosome transfer. Indeed, miRs have been widely recognized for their crucial roles in the regulation of gene expression through targeting the 3′UTR of downstream genes, and it is known that one miR can regulate hundreds of different genes [18,19]. Based on our results, we hypothesized that TBX2-mediated miR regulation may be responsible for both cell autonomous (intracellular) and non cell-autonomous (intercellular) regulation of NEPC transdifferentiation. We therefore performed an unbiased next-generation sequencing (NGS) analysis of exosomes derived from PC3^TBX2DN^ or PC3^Neo^ cells in an effort to identify TBX2-regulated miRs as depicted in Figure 2A. The differential expression of miRs (top 20) in PC3^TBX2DN^ cells when compared with PC3^Neo^ cells is shown in Figure 2B. Further, we analyzed the targets of the top five upregulated and top five downregulated miRs of the NGS analysis (Figure 2C). Because (a) miR-200c-3p has been reported to be decreased in human CRPC [37,38,39] and (b) to negatively regulate SOX2 expression [23,24,39,40,41] and (c) our in vitro data showed that SOX2 and N-MYC were consistently altered upon TBX2 modulation, we prioritized miR-200c-3p for further study. In silico analysis (using miRDB and Targetscan) was utilized to predict the probable binding sites for miR-200c-3p in the 3′ UTRs of MYCN and SOX2 genes (Figure 2D). Quantitative real-time RT-PCR (qRT-PCR) was performed to confirm the results of the NGS analysis. We found that while miR-200c-3p expression was dramatically upregulated in PC3^TBX2DN^ and C4-2B^TBX2DN^ cells and in the exosomes derived from these cells when compared with the respective Neo controls, the converse approach of TBX2 overexpression in LNCaP cells led to downregulated miR-200c-3p expression in these cells as well as in the exosomes obtained from these cells (Figure 2E,F). These results suggest that miR-200c-3p is a downstream mediator of TBX2 signaling, and that TBX2/miR-200c-3p/SOX2/N-MYC signaling axis has an important role in NEPC transdifferentiation.

We next sought to determine if TBX2 directly regulates miR-200c-3p. In silico analysis was performed to identify potential TBX2 binding sites in the promoter region of this miR. Based on the published consensus TBX2 binding sequence [42] and also due to similarity with Brachyury [43], we identified multiple TBX2-binding sites in the promoter region of miR-200c-3p (Figure 2G). ChIP assays showed that TBX2 binds directly to two sites within the miR-200c-3p promoter region located upstream to the transcription start site (−681, and −931 bp, Figure 2H) and showed ~8-fold and 6-fold of TBX2 occupancy, respectively, when analyzed by qRT-PCR after normalization by input (Figure 2I). As TBX2 has been reported to primarily function as a transcriptional repressor [44,45], these results strongly suggest direct regulation of miR-200c-3p in PCa by TBX2 through transcriptional repression.

### 3.3. Abrogated Metastasis in a Mouse Model Caused by Blocking Endogenous TBX2 Results in the Upregulation of miR-200c-3p and Downregulation of SOX2/N-MYC

**Figure 3:**
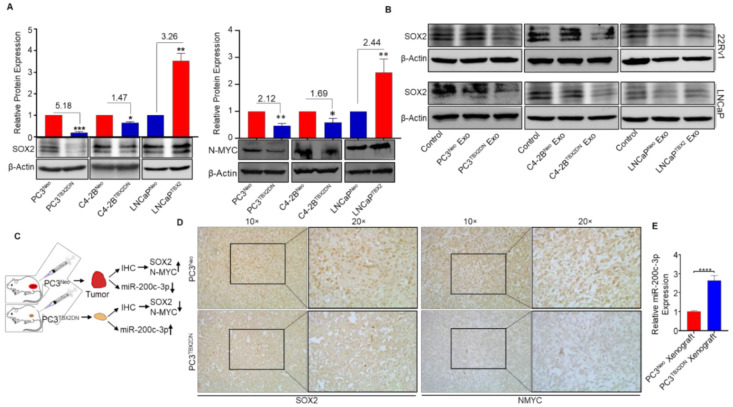
Blocking TBX2 in an in vivo mouse model of PCa progression and metastasis leads to increased miR-200c-3p and reduced SOX2 and N-MYC expression. (**A**) Western blots showing SOX2 and N-MYC expression in TBX2-modulated PCa cells; (**B**) Western blots showing SOX2 expression in LNCaP or 22Rv1 cells treated with exosomes (20 μg/mL, added a total of two times on alternate days) isolated from TBX2-modulated human prostate cancer (PCa) cells. Densitometric analysis of the Western blots is provided in Figure S4; (**C**) a schematic of the orthotopic xenograft experiment; (**D**) immunohistochemical (IHC) staining showing SOX2 and N-MYC expression in the orthotopic xenografts of PC3^Neo^ or PC3^TBX2DN^ human PCa cells. Densitometric analysis of the IHC images is provided in Figure S5; (**E**) miR-200c-3p expression in the orthotopic xenografts of PC3^Neo^ or PC3^TBX2DN^ cells using quantitative real-time RT-PCR analysis (qRT-PCR). Data represent the average of triplicates values ± S.D.; Student’s unpaired 2-tailed *t*-tests were performed to compare the two groups, *, *p* ≤ 0.05; **, *p* < 0.01, and ***, *p* ≤ 0.001, ****, *p* ≤ 0.0001. The uncropped Western blot images can be found in Figures S8–S10.

As our results thus far pointed to SOX2 and N-MYC as being regulated by TBX2/miR-200c-3p signaling, we examined their expression in our in vitro and in vivo model systems. A recent bioinformatics-based report using NEPC datasets identified a 12-gene human NEPC signature including SOX2 [34]. Further, SOX2 has been reported to promote CRPC and lineage plasticity resulting in transdifferentiation to NEPC [9,10,34,46]. In addition, N-MYC has been reported to drive NEPC [11,13]. Western blot analysis showed that while SOX2 and N-MYC expression were significantly reduced in PC3^TBX2DN^ and C4-2B^TBX2DN^ cells; conversely, SOX2 and N-MYC expression were significantly increased in LNCaP^TBX2^ when compared with the respective Neo controls (Figure 3A). Further, consistent with the corresponding mRNA data (Figure 1K–P), the treatment of LNCaP or 22Rv1 human PCa cells with exosomes derived from TBX2DN cells (PC3^TBX2DN^ or C4-2B^TBX2DN^) decreased SOX2 expression, while conversely, exosomes obtained from LNCaP^TBX2^ cells increased SOX2 expression when compared with the exosomes obtained from the respective Neo control cells (Figure 3B).

We previously reported that PC3^TBX2DN^ xenografts (depicted in Figure 3C) display reduced local invasion and abrogated metastatic ability to the regional lymph nodes when compared with xenografts from the control PC3^Neo^ cells [26]. Consistent with the in vitro results, immunohistochemical analysis of the PC3^TBX2DN^ orthotopic xenografts displayed reduced SOX2 and N-MYC expression when compared with control PC3^Neo^ xenografts (Figure 3D). Further, miR-200c-3p was elevated in PC3^TBX2DN^ xenografts when compared with the Neo controls (Figure 3E). Altogether, these in vivo results supported our in vitro findings that miR-200c-3p, SOX2, and N-MYC are downstream of TBX2 signaling, and that while SOX2 and N-MYC display a positive relation with TBX2, miR-200c-3p shows an inverse relation with TBX2.

### 3.4. miR-200c-3p Is the Intermediary Effector in TBX2 Regulation of SOX2 and MYCN

**Figure 4:**
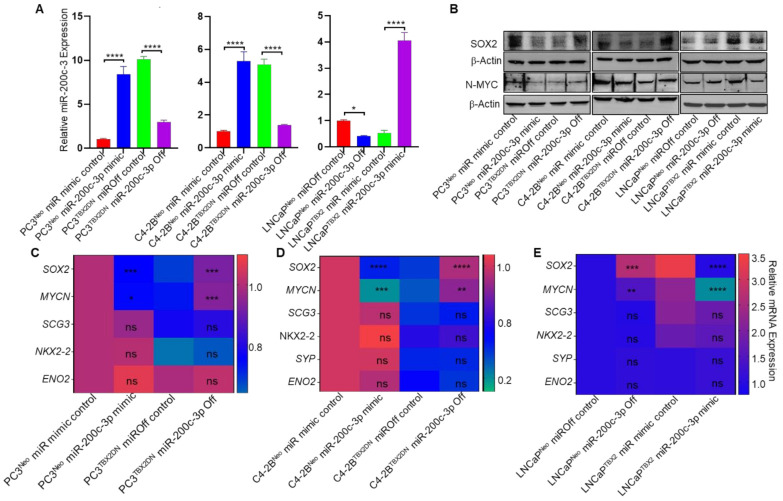
Alteration of miR-200c-3p expression in the context of TBX2 modulation rescues SOX2 and MYCN. (**A**) Quantitative real-time RT-PCR (qRT-PCR) analysis showing the validation of the approaches for miR-200c-3p modulation [following miR-200c-3p off (knockdown) or miR-200c-3p mimic (overexpression)] in human PCa cells; (**B**) Western blots showing SOX2 and N-MYC expression following the rescue of miR-200c-3p expression in the context of TBX2 genetic modulation. Densitometric analysis is provided in Figure S6; (**C**–**E**) heatmap summarizing the qRT-PCR results comparing the expression of neuroendocrine markers following miR-200c-3p rescue approaches in TBX2-modulated human PCa cells. Data are represented as mean ± SD (*n* = 3), Student’s unpaired 2-tailed *t*-tests were performed to compare the two groups or one-way ANOVA for more than 2 groups. *, *p* ≤ 0.05; **, *p* < 0.01; ***, *p* <0.001; ****, and *p* < 0.0001, and ns indicates not significant. The uncropped Western blot images can be found in Figure S11.

To elucidate the role of miR-200c-3p in TBX2/miR-200c-3p/SOX2/N-MYC signaling, we rescued miR-200c-3p levels in human PCa cells in the context of TBX2 genetic modulation. For this experiment, two separate approaches were used. First, we stably knocked down miR-200c-3p in PC3^TBX2DN^, C4-2B^TBX2DN^ and LNCaP^Neo^ cells that showed high miR-200c-3p expression. Second, we stably overexpressed miR-200c-3p in PC3^Neo^, C4-2B^Neo^, and LNCaP^TBX2^ cells that showed decreased miR-200c-3p expression. Expression analysis of miR-200c-3p confirmed the successful establishment of these models (Figure 4A). Expression analysis showed that miR-200c-3p knockdown in PC3^TBX2DN^ and C4-2B^TBX2DN^ restored SOX2 and MYCN, while activation of miR-200c-3p in LNCaP cells repressed SOX2 and MYCN at the protein (Figure 4B) and mRNA levels (Figure 4C–E). These results strongly point to TBX2/miR-200c-3p signaling as the upstream mediator of SOX2 and MYCN in PCa.

### 3.5. TBX2 Is Associated with SOX2 and MYCN in Human PCa

**Figure 5:**
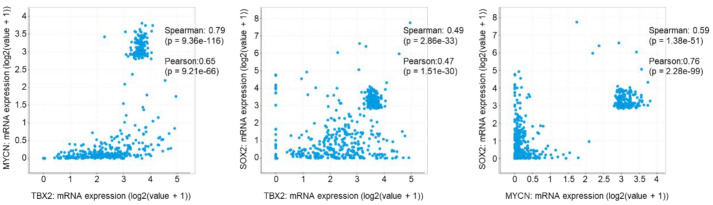
TBX2 is associated with MYCN and SOX2 in human PCa samples. Plots showing pair-wise correlations between the mRNA expression of TBX2, MYCN, and SOX2 in 531 PCa samples (c-bioportal) [32,33].

Based on our in vitro and in vivo data, we checked the status of TBX2, MYCN, and SOX2 in publicly available data sets of human PCa. An analysis of 531 human PCa samples using the c-bioportal database [32,33] revealed: (a) a strong positive correlation between TBX2 and MYCN (Spearman 0.79, *p* = 9.36 × 10^−^^116^), (b) a moderate positive correlation between TBX2 and SOX2 (Spearman 0.49, *p* = 2.86 × 10^−33^), and (c) a strong positive correlation between MYCN and SOX2 (Spearman 0.59, *p* = 1.38 × 10^−51^) (Figure 5). Therefore, consistent with our in vitro and in vivo studies, these data point to the TBX2/SOX2/N-MYC signaling axis in human PCa.

## 4. Discussion

Deciphering the signaling mechanisms that drive t-NEPC/NEPC transdifferentiation is vital to understanding this pathophysiology and in developing novel therapeutic modalities against this aggressive subtype of PCa. Despite progress in the recent years in identifying a few discrete molecular drivers of t-NEPC/NEPC transdifferentiation including SOX2 and N-MYC [6,7,8,9,10,11,12,13], fundamental questions remain that could provide critical clues to the NEPC phenomenon. For instance, the molecular mechanisms/signaling events that drive t-NEPC/NEPC transdifferentiation remain largely unknown. In addition, how NEPC foci communicate with the neighboring adenocarcinoma/CRPC cells to further propagate the NEPC phenotype remains unresolved. It is within this backdrop of progression to advanced PCa that our results are of critical significance.

**Figure 6:**
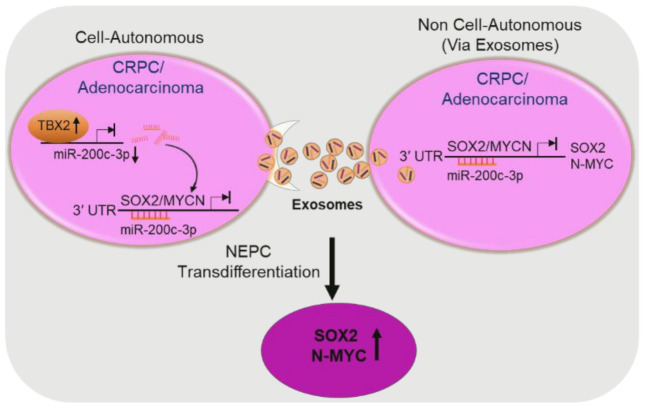
Schematic of the proposed mechanism of TBX2/miR-200c-3p/SOX2/N-MYC signaling in the progression from CRPC to NEPC. TBX2 upregulation in PCa adenocarcinoma/CRPC cells results in the repression (down-headed arrow) of miR-200c-3p via direct binding to its promoter. miR-200c-3p in turn—either through a cell-autonomous mode, or through a non cell-autonomous mode via exosomes—mediates the repression of SOX2 and MYCN. The resultant net effect is that TBX2 upregulation in PCa leads to the upregulation (up-headed arrow) of SOX2 and N-MYC, and the propagation of the SOX2- and N-MYC- driven NEPC phenotype.

Our study identifies the TBX2/miR 200c-3p axis as a critical upstream regulator of SOX2 and N-MYC—two established drivers of NEPC transdifferentiation [9,10,11,13] (Figure 6). Further, the unbiased identification of miR-200c-3p as a downstream effector of TBX2 (Figure 2B) juxtaposed with the miR-200c-3p rescue experiments in the context of TBX2 genetic modulation (Figure 4A–E) reveals a hitherto little known but a central biological function of miR-200c-3p as a crucial mediator of TBX2 signaling in driving the NEPC phenotype. Our study sheds light on the dual level of control exerted by TBX2/miR-200c-3p signaling in mediating SOX2/N-MYC driven NEPC pathophysiology, i.e., via: (a) cell-autonomous intracellular gene expression changes and (b) non cell-autonomous intercellular paracrine communication via exosomes.

The relevance of our findings that point to this dual mode of action of the TBX2/miR-200c-3p/SOX2/N-MYC signaling axis in NEPC transdifferentiation is in agreement with other observations in this space. For example, interspersed foci with neuroendocrine marker expression within the backdrop of CRPC is observed in pathologic specimens. In addition, paracrine factors secreted by NEPC cells have the potential to interact with surrounding CRPC cells to further propagate the NEPC phenotype [14,47].

Our findings are corroborated by previous reports that have elucidated the role of EVs (exosomes) as a delivery mechanism of cancer cells and have implicated them in diverse facets of tumor progression and metastatic manifestation [14,15,17,21,27]. Intriguingly, a pioneering study had shown that factors secreted by NEPC have the potential to promote the growth of androgen dependent LNCaP tumors to grow in castrated mice [47]. The role of exosomes in PCa pathophysiology was appreciated in subsequent investigations; however, the contribution of exosomes in fostering the molecular mechanisms that orchestrate the NEPC phenotype remains largely elusive. Interestingly and in contrast with other EV fractions (Figures S1 and S2), our initial experiments suggested that TBX2 exerts its effects primarily through exosomes; therefore, our studies focused on this component of the EVs.

Previous reports have indicated that miRs are abundantly present in exosomes, and exosomes have drawn increasing attention due to their ability to regulate many pathways during tumor progression [21]. In light of the pathophysiology of NEPC, our hypothesis that a miR could be the mediator of TBX2 signaling in NEPC transdifferentiation is borne out of numerous reports that have established miRs as crucial regulators of gene expression at both the intracellular and intercellular levels [18,19,21]. miR-200c-3p, one of the top miRs that was dysregulated in the exosomes derived from TBX2DN cells, particularly caught our attention because of its reported loss in CRPC [37,48]. This suggested that miR-200c-3p could be a downstream signaling mediator of TBX2’s action in CRPC progression to NEPC. In addition to identifying the presence of miR-200c-3p binding sites on 3′UTRs of SOX2 and N-MYC, our experiments show that miR-200c-3p rescue in the context of TBX2 modulation leads to SOX2/MYCN rescue (Figure 4B–D). In addition, we identify the direct binding of TBX2 on the miR-200c-3p promoter. Taken together, these results strongly point to miR-200c-3p as a critical mediator of TBX2/SOX2/N-MYC signaling axis in NEPC transdifferentiation.

Our research group was the first to report elevated expression of TBX2 in CRPC and showed that TBX2 in primary PCa mediates multiple steps of the metastatic cascade [26]—pointing to its key role in disease progression. In agreement with our findings, a recent report established TBX2 as one of the four transcription factors in the context of CHD1 loss that drive transcriptional plasticity resulting in antiandrogen resistance in CRPC [49]. In addition, a recent bioinformatics-analysis-based report of publicly available NEPC datasets identified TBX2 as one of the key upstream regulators of several commonly upregulated genes in human NEPC [34]. The findings in our present study unravel an additional layer to TBX2’s key role in the progression to advanced PCa and in particular provide mechanistic insights into the progression of CRPC to NEPC. Although not in the scope of the present study, identifying additional targets of TBX2/miR-200c-3p signaling will provide important information regarding how TBX2 regulates the transcriptome and point to other crucial effectors of the TBX2/miR-200c-3p pathway that drive NEPC transdifferentiation. These studies could lead to valuable insights towards identifying novel therapeutic targets for the treatment of the disease progression from CRPC to NEPC.

Based on our previous study [26], we found the loss-of-function studies as ideal for blocking TBX2, and hence, we used this approach for the present study. Further, the repressive function of TBX2 in this study is in line with previous reports that have focused on TBX2 repression of its effectors [42,44,45]. Although the TBX2 protein contains both the activation and repression domains, TBX2 has predominantly been reported to function as a transcriptional repressor [44]. The TBX2 DN mutant construct contains the T-box DNA-binding domain but lacks the carboxy-terminal residues necessary for transcriptional repression [50]—thereby making it an ideal approach to specifically investigate transcriptional repression. In addition, previous reports including our study had found that TBX2DN works in congruence with the RNA interference approach and upregulates p21, a known TBX2 target [26,45,50].

Finally, although the present study was focused on the role of exosomal miR-200c-3p in promoting the NEPC phenotype between neighboring PCa cells, in our orthotopic mouse model of PCa metastasis, we observed increased expression of miR-200c-3p in the human TBX2DN PCa xenografts that display abrogated metastatic ability to the lymph nodes (compared with Neo controls) (Figure 3E). This opens up an intriguing question if TBX2/miR-200c-3p/SOX2/N-MYC signaling could potentially drive metastatic manifestation at the secondary sites via exosomal transfer. The insights offered by these investigations could provide further clues into the NEPC transdifferentiation puzzle especially in lieu of our previous report that delineated the role of TBX2 in multiple facets of PCa progression including distant metastasis [26].

As research on the clinical challenges posed by potent ADTs is garnering increasing recognition, the emphasis on discovering key drivers of t-NEPC/NEPC transdifferentiation is gaining momentum, and the list of key drivers keeps increasing [6,7,8,9,10,11,12,13]. The goal of these studies including ours is to improve PCa therapy through advancing our understanding of the molecular effectors/signaling pathways that orchestrate t-NEPC/NEPC transdifferentiation as a mechanism of acquired therapeutic resistance.

## 5. Conclusions

Our study has identified a novel mechanism wherein TBX2 drives NEPC transdifferentiation via miR-200c-3p/SOX2/N-MYC signaling. Further, our investigations point to positive correlations between TBX2 and SOX2/N-MYC expression in human PCa patient samples. Our findings may pave the way for the development of novel and effective therapeutic strategies against the progression from CRPC to NEPC through targeting the TBX2/miR-200c-3p/SOX2/N-MYC axis.

## Data Availability

All the data used and analyzed in the current study are available from the corresponding authors on reasonable request.

## Supplementary Materials

The following information is available online at https://www.mdpi.com/article/10.3390/cancers13195020/s1, Figure S1: Larger extracellular vesicles [such as apoptotic bodies (ABs), microvesicles (MVs)] or soluble factors (SFs) did not affect the expression of neuroendocrine markers in LNCaP cells, Figure S2: Larger extracellular vesicles [such as apoptotic bodies (ABs), microvesicles (MVs)] or soluble factors (SFs) did not affect the expression of neuroendocrine markers in 22Rv1 cells, Figure S3: Magnified image of Figure 2C, Figure S4: Densitometric analysis of the Western blot images in Figure 3B, Figure S5: Densitometric analysis using Fiji image J2 for IHC images in Figure 3D, Figure S6: ImageJ-based densitometric analysis for (A) SOX2, and (B) N-MYC for the Western blot images in Figure 4B, Figure S7: Uncropped Western blot images for Figure 1H, Figure S8: Uncropped Western blot images for Figure 3A, Figure S9: Uncropped Western blot images for Figure 3B top panel, Figure S10: Uncropped Western blot images for Figure 3B bottom panel, Figure S11: Uncropped Western blot images for Figure 4B, Table S1: List of the primers used for quantitative real-time RT-PCR (qRT-PCR) analyses performed in this study, Table S2: List of the primers used for chromatin immunoprecipitation (ChIP) analyses performed in this study, and Table S3: List of the antibodies used in this study.

## Abbreviations

AB/ABs	Apoptotic bodies
ANOVA	Analysis of Variance
ARF6	ADP Ribosylation Factor 6
ASH1	Absent Small and Homeotic Disks Protein 1 Homolog
AURKA	Aurora Kinase A
C4-2B^TBX2DN^	Genetic modulation of endogenous TBX2 in C4-2B human prostate cancer cells using the Dominant Negative (DN) construct
CD	Cluster of Differentiation
CHG	Chromogranin
CRPC	Castrate Resistant Prostate Cancer
DAPI	4,6-diamino-2-phenylindole
ENO2	Enolase 2
Exo	Exosomes
FITC	Fluorescein isothiocyanate
hsa-miR	human microRNA
IACUC	Institutional Animal Care and Use Committee
IgG	Immunoglobulin G
IHC	Immunohistochemistry
ires GFP	Internal Ribosome Entry Sites Green Fluorescence Protein
KEGG	Kyoto Encyclopaedia of Genes and Genomes
LNCaP^TBX2^	Genetic modulation of TBX2 in LNCaP human prostate cancer cells using the over-expression construct
miR	microRNA
Ms	Mouse
MV/MVs	Microvesicles
NCAM1	Neural cell adhesion molecule1
NE	Neuroendocrine
Neo	Control Vector
NEPC	Neuroendocrine Prostate Cancer
NGS	Next Generation Sequencing
NKX2-2	NK2 HomeoBox 2
N-MYC (encoded by MYCN)	Neuroblastoma-Derived V-Myc Avian Myelocytomatosis Viral Related Oncogenic protein
PCa	Prostate Cancer
PC3^TBX2DN^	Genetic modulation of endogenous TBX2 in PC3 human prostate cancer cells using the Dominant Negative (DN) construct
qRT-PCR	Real-Time Quantitative Reverse Transcription Polymerase Chain Reaction
RT	Room temperature
SCG3	Secretogranin III
SD	Standard deviation
SF/SFs	Soluble factors
SOX2	SRY-Box transcription Factor 2
SYP	Synaptophysin
TBX2	T-Box transcription Factor 2
T.D	Transmitted Detector
THBS1	Thrombospondin 1
t-NEPC	Treatment-induced Neuroendocrine Prostate Cancer
UTR	UnTranslated Region
