# Supplementary material for: TBX2 Drives Neuroendocrine Prostate Cancer through Exosome-Mediated Repression of miR-200c-3p
Source: Cancers (Basel). 2021 Oct 7;13(19):5020. doi: 10.3390/cancers13195020 (PMC8507954; doi:10.3390/cancers13195020)
Supplement: Supplementary file 1 [file cancers-13-05020-s001.zip › cancers-1349342-supplementary.pdf]

Supplementary Materials

TBX2 Drives Neuroendocrine Prostate Cancer through Exosome-Mediated Repression of miR-200c-3p

Girijesh Kumar Patel, Sayanika Dutta, Mosharaf Mahmud Syed, Sabarish Ramachandran, Monica Sharma, Venkatesh Rajamanickam, Vadivel Ganapathy, David J. DeGraff, Kevin Pruitt, Manisha Tripathi, and Srinivas Nandana

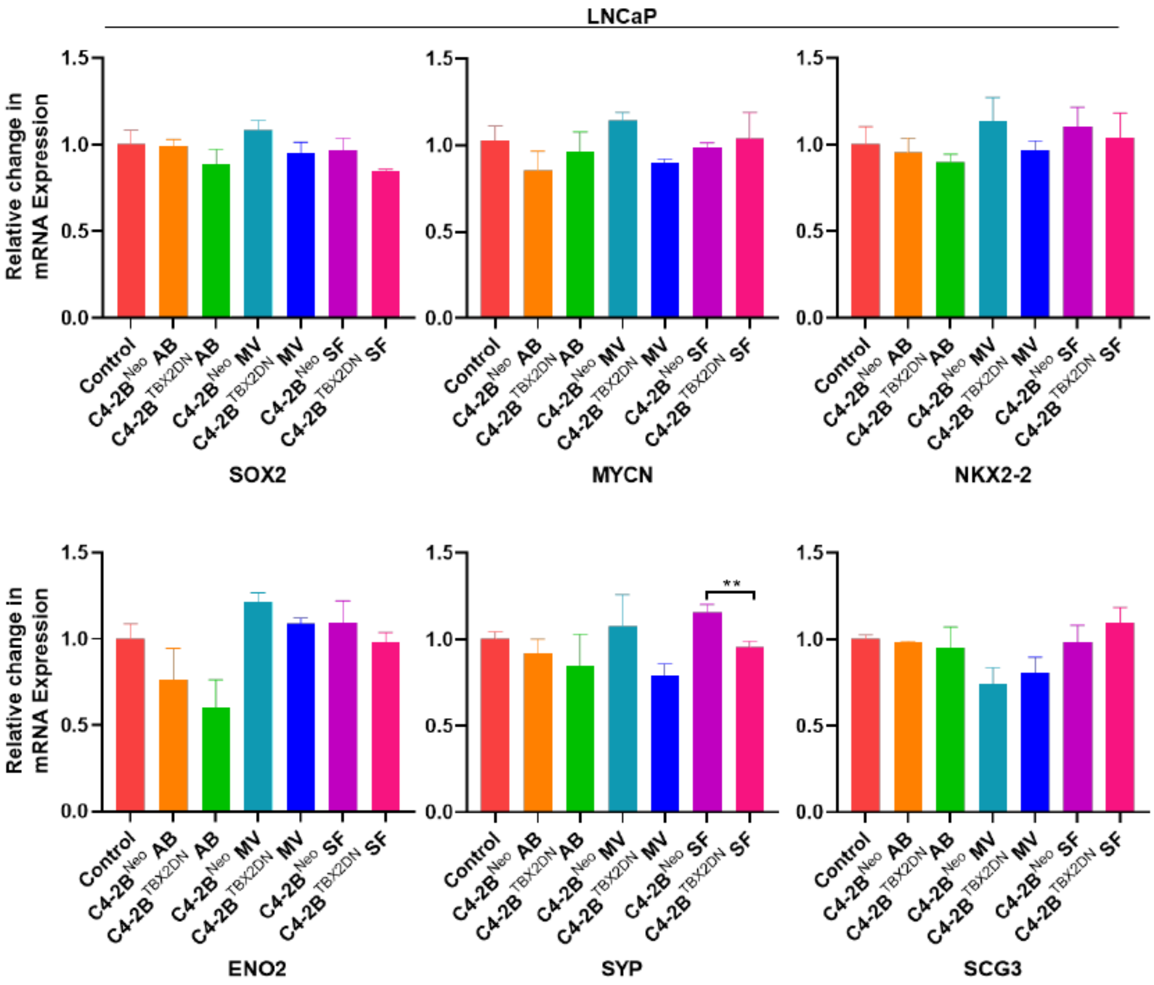

Figure S1. Larger extracellular vesicles [such as apoptotic bodies (ABs), microvesicles (MVs), and soluble factors (SF)] did not affect the expression of neuroendocrine (NE) markers in LNCaP cells. All the extracellular vesicles [such as apoptotic bodies (ABs), microvesicles (MVs), exosomes, and soluble factors (SF) were fractionated from the culture media of TBX2-modulated PCa cells. LNCaP cells were treated with of ABs or MVs (20 µg/mL) or SF on alternate days for a total of two times and cells were harvested on the fourth day. RNA was isolated, complementary DNA (cDNA) synthesized, and quantitative real-time PCR (qRT-PCR) analyses were performed to screen the expression of NE markers). Data are represented as mean ± SD (n = 3), Student's unpaired 2-tailed t-tests were performed to compare the two groups, **, $p < 0.01$.

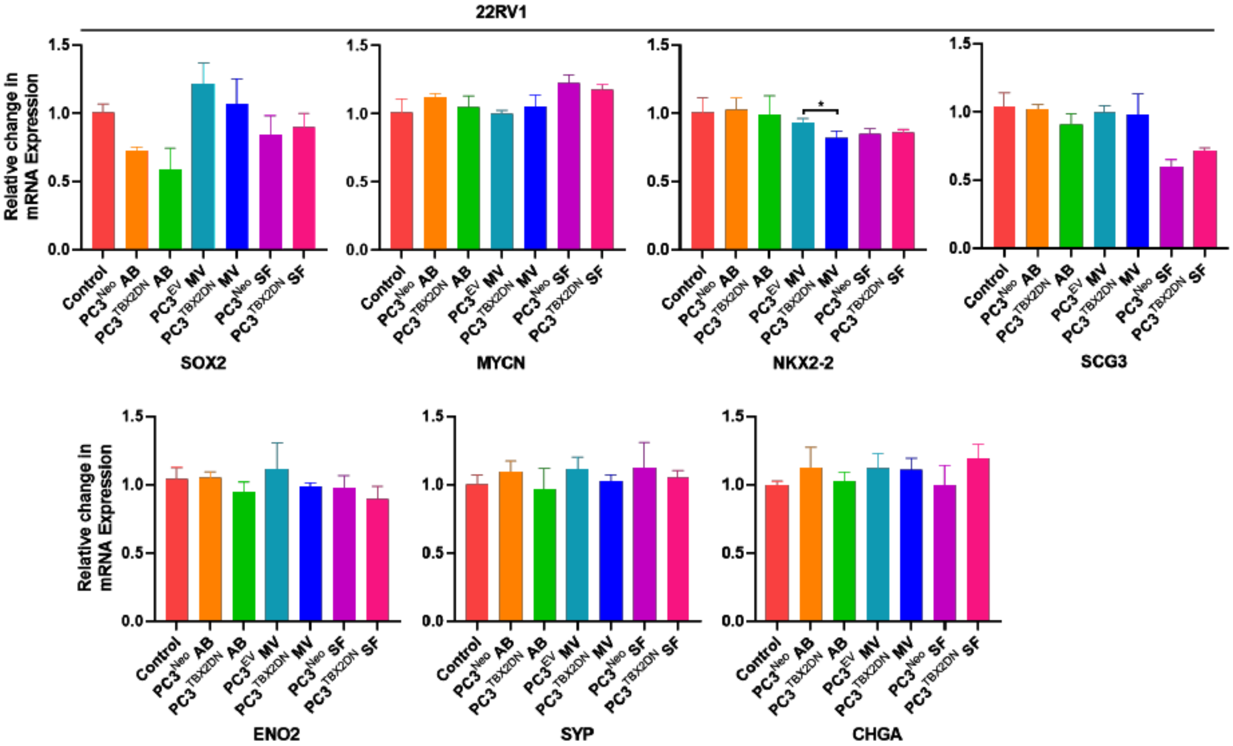

Figure S2. Larger extracellular vesicles (such as apoptotic bodies (ABs), microvesicles (MVs), and soluble factors (SF)) did not affect the expression of neuroendocrine (NE) markers in 22Rv1 cells. All the extracellular vesicles (such as apoptotic bodies (ABs), microvesicles (MVs), exosomes), and soluble factors (SF) were fractionated from the culture media of TBX2-modulated cells. 22Rv1 cells were treated with of ABs or MVs (20 µg/mL) or SF on alternate days for a total of two times and the cells were harvested on the fourth day. RNA was isolated, complementary DNA (cDNA) synthesized, and quantitative real-time PCR (qRT-PCR) were performed to screen the NE marker expression). Data are represented as mean ± SD ($n = 3$), Student's unpaired 2-tailed t-tests were performed to compare between the two groups. We did not find significant changes between the two groups i.e., control (Neo) vs. TBX2DN groups.

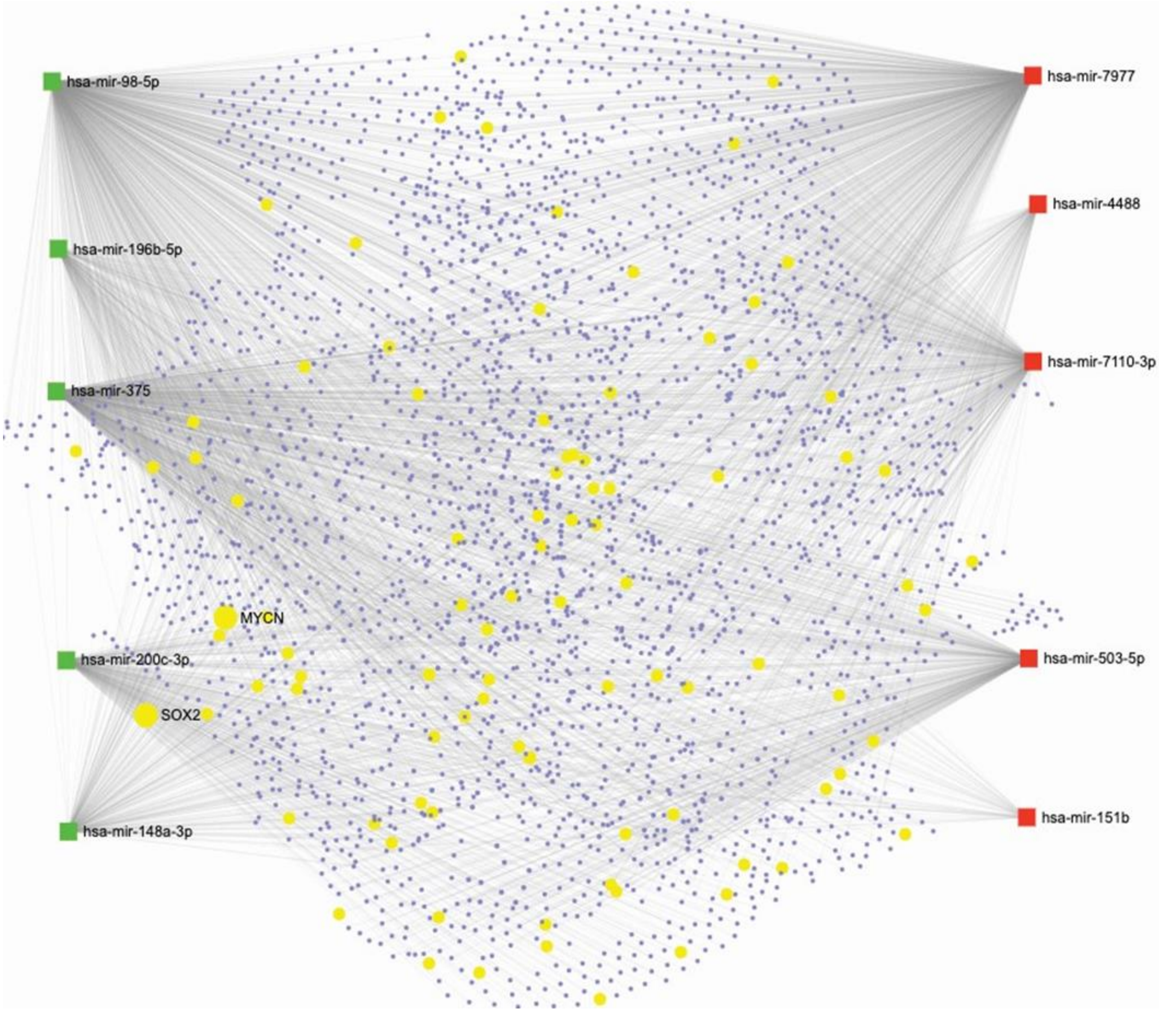

Figure S3. Magnified image of Figure 2C. miRNET2.0-based analysis showing the interactions amongst the top 5 upregulated microRNA (miRs) (as green squares) and the top 5 downregulated miRs (as red squares) in the exosomes derived from PC3^{TBX2DN} cells compared with PC3^{Neo} cells. The circular nodes in yellow represent the genes that are enriched in neuronal pathways as found in the Kyoto Encyclopedia of Genes and Genomes (KEGG) and reactome databases. The circular nodes in steel blue represent all the other target genes for these differentially expressed miRs.

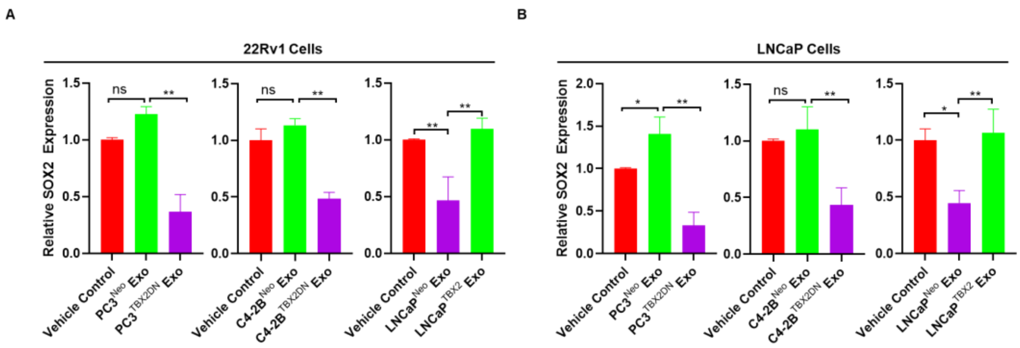

Figure S4. Densitometric analysis for the Western blot images in Figure 3B. (**A**) SOX2 expression in 22Rv1 cells treated with exosomes (Exo) derived from TBX2 modulated PCa cells or vehicle control; (**B**) SOX2 expression in LNCaP cells treated with exosomes derived from TBX2 modulated PCa cells or vehicle control. Data are represented as mean ± SD (n = 3), one-way ANOVA was performed where (n = 3), ns, not significant, *, $p < 0.05$, and **, $p < 0.01$.

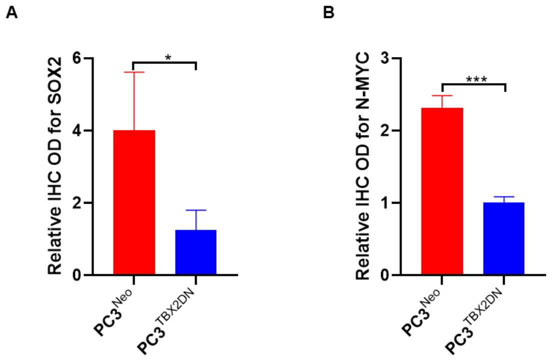

Figure S5. Densitometric analysis using Fiji image J2 for immunohistochemistry (IHC) images in Figure 3D. (**A**) Relative optical density (OD) of immunohistochemical staining for SOX2; (**B**) relative OD of immunohistochemical staining for N-MYC. Data are represented as mean ± SD ($n = 3$), Student's unpaired 2-tailed t-tests were performed to compare between the two groups. *, $p < 0.05$; ***, $p < 0.001$.

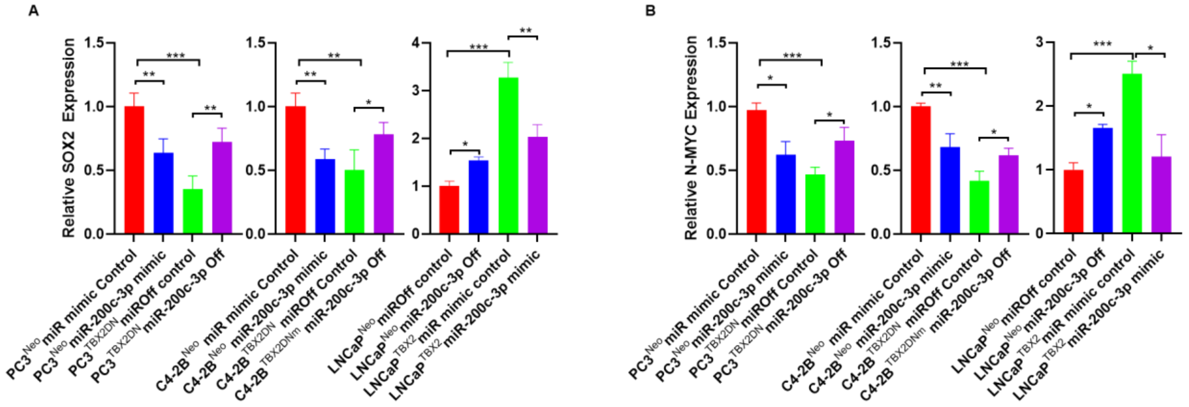

Figure S6. ImageJ-based densitometric analysis of (**A**) SOX2, and (**B**) N-MYC for the Western blot images in Figure 4B following microRNA-200c-3p (miR-200c-3p) rescue. Data are represented as mean ± SD ($n = 3$), one-way ANOVA was performed where ($n = 3$), *, $p < 0.05$; **, $p < 0.01$; and ***, $p < 0.001$.

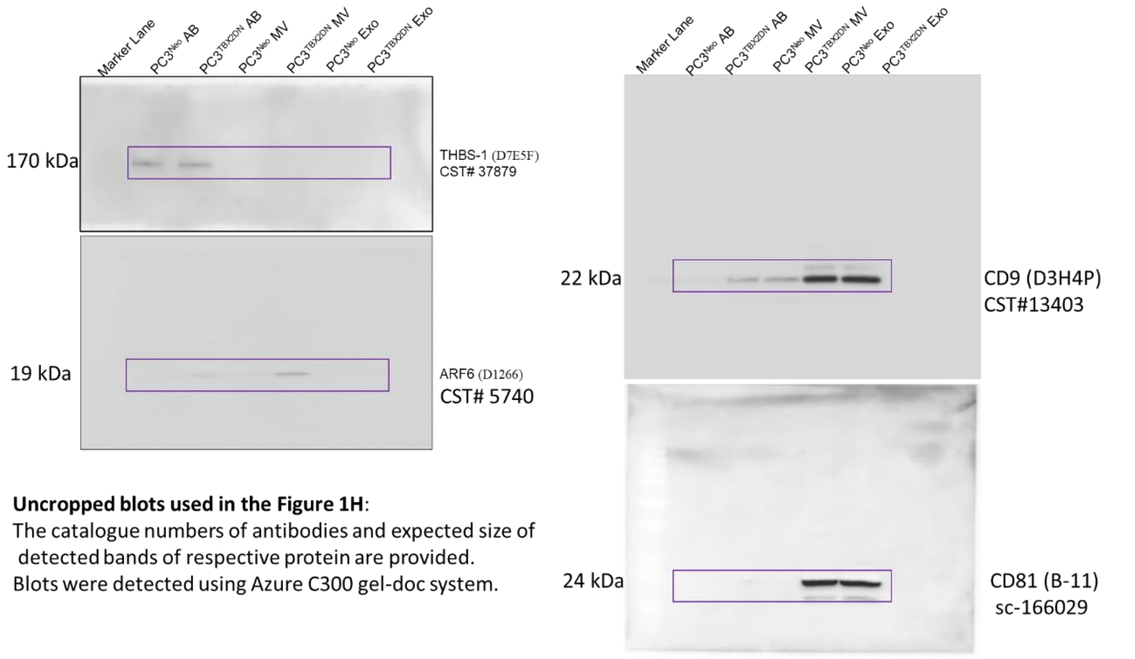

Figure S7. Uncropped Western blot images for Figure 1H. AB (apoptotic bodies), MV (microvesicles) and Exo (exosomes).

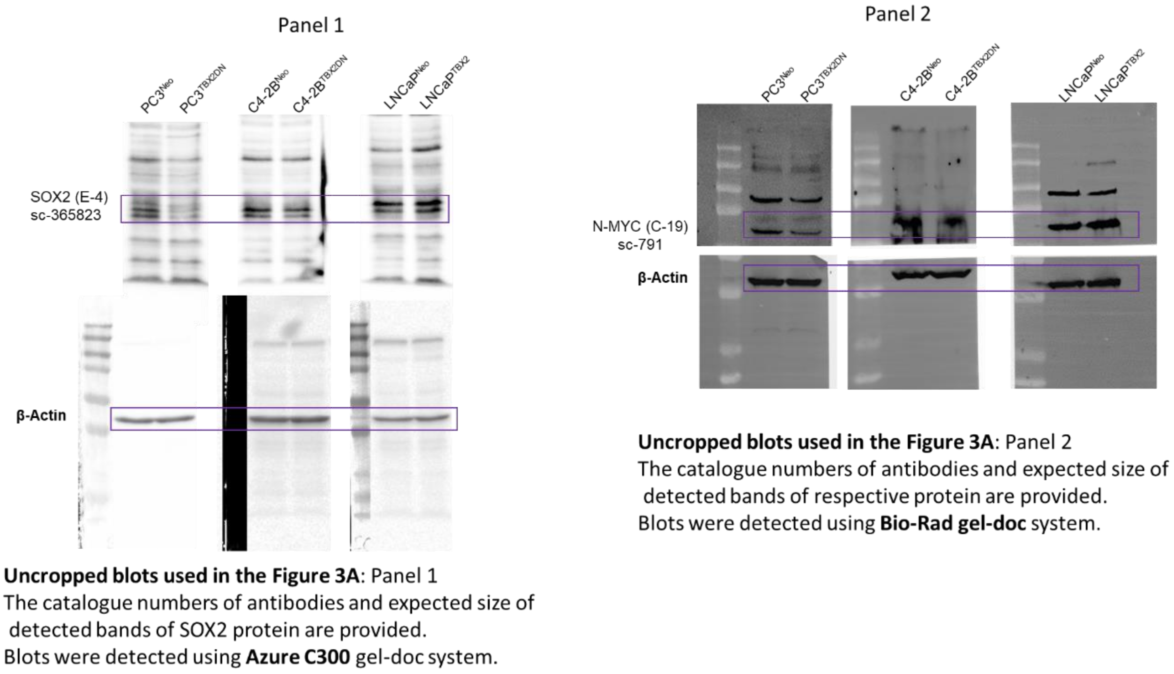

Figure S8. Uncropped Western blot images for Figure 3A. (**Left Panel**) Western blot for SOX2. (**Right Panel**) Western blot for N-MYC.

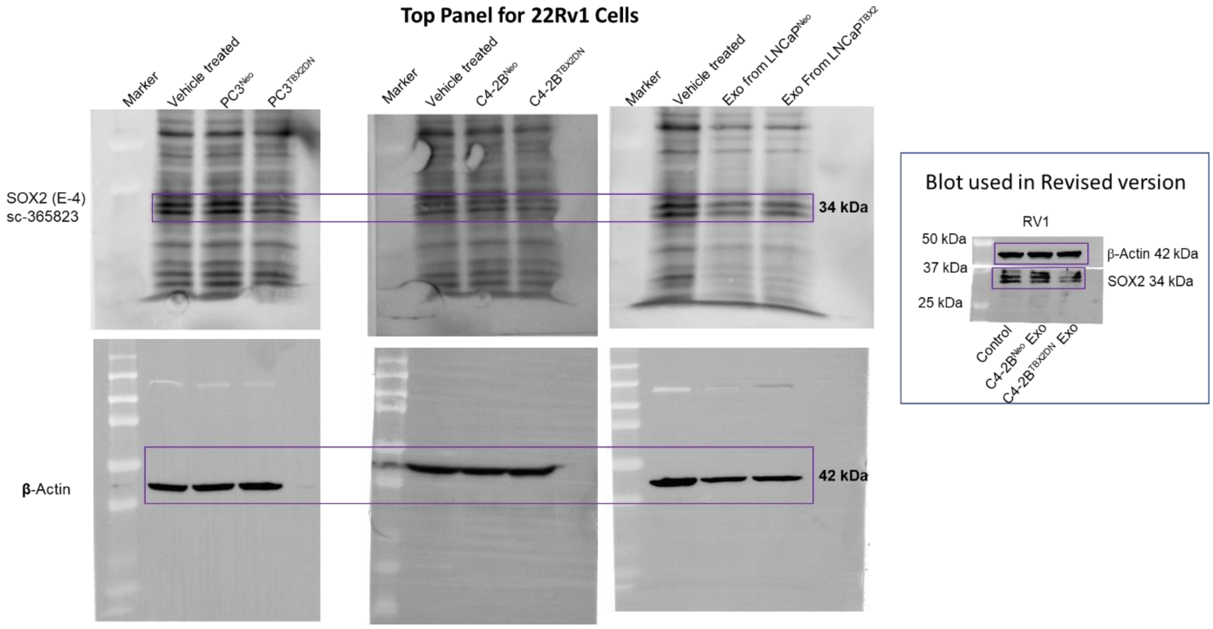

Figure S9. Uncropped Western blot images for Figure 3B top panel. 22Rv1 cells were treated with vehicle control or exosomes (Exo) isolated from TBX2-modulated PCa cells.

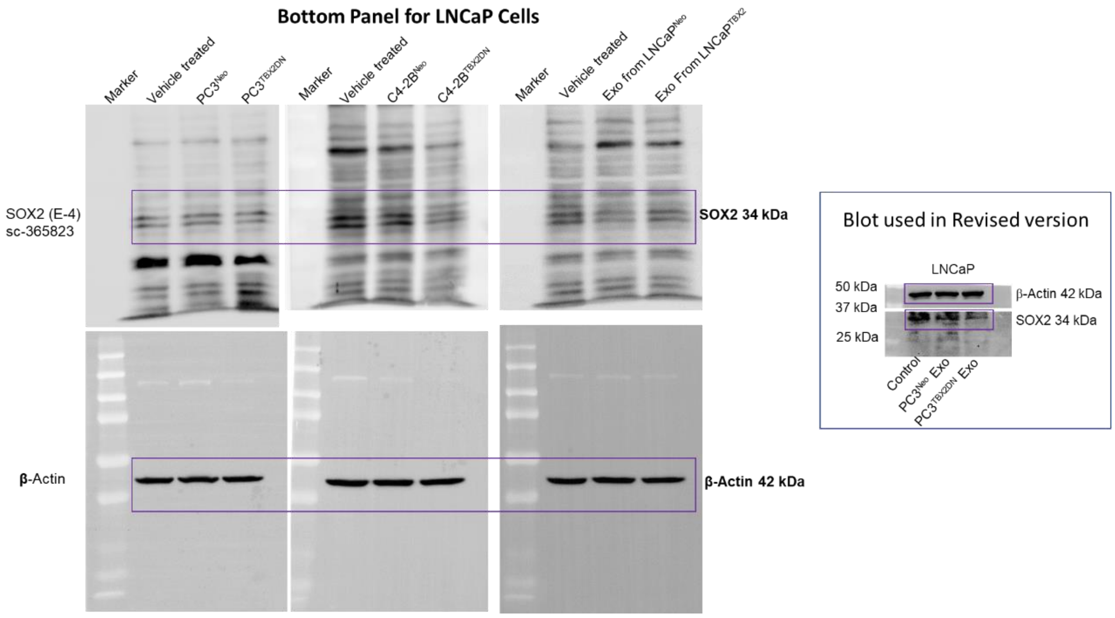

Figure S10. Uncropped Western blot images for Figure 3B bottom panel. LNCaP cells were treated with vehicle control or exosomes (Exo) isolated from TBX2-modulated PCa cells.

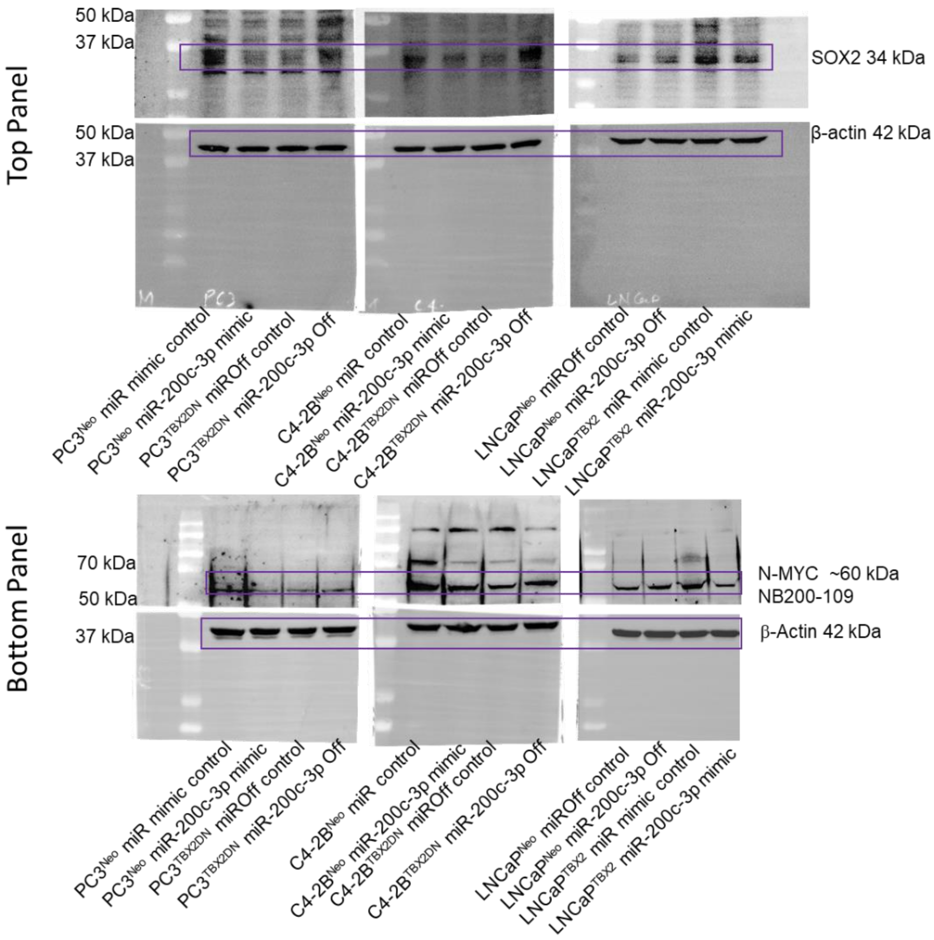

Figure S11. Uncropped Western blot images for Figure 4B. Top Panel shows Western blot for SOX2. Bottom Panel shows Western blot for N-MYC.

Table S1. List of quantitative real-time PCR (qRT-PCR) primers used in the study.

Gene Name	Primer Sequence 5′ to 3′
hSOX2-110 F	TACAGCATGTCCTACTCGCAG
hSOX2-110 R	GAGGAAGAGGTAACCACAGGG
hSYP-122 F	CTCGGCTTTGTGAAGGTGCT
hSYP-122 R	CTGAGGTCACTCTCGGTCTTG
hCHGA-75 F	CTCCCTGTGAACAGCCCTA
hCHGA-75 R	TGTGTCGGAGATGACCTCAA
hENO2-149 F	CCGGGAACTCAGACCTCATC
hENO2-149 R	CTCTGCACCTAGTCGCATGG
hSCG3-164 F	ATTCTGGTGTTAGTGCTCCCG
hSCG3-164 R	GGCTTGTTTTCTGGAGGATATGT
hCHGB-133 F	CGAGGGGAAGATAGCAGTGAA
hCHGB-133 R	CAGCATGTGTTTCCGATCTGG
hMYCN-113 F	TGATCCTCAAACGATGCCTTC
hMYCN-113 R	GGACGCCTCGCTCTTTATCT
hNKX2-2-121 F	ACAACTGGTGGCAGATTTCG
hNKX2-2-121 R	CCCAAACAAGCCACAAAGAA
hAURKA-205 F	GAGGTCCAAAACGTGTTCTCG
hAURKA-205 R	ACAGGATGAGGTACACTGGTTG
hNCAM1-124 F	GGAACCCAGTGCACCTAAGCT
hNCAM1-124 R	CGCTCGGTACCTGACCAGAT
hASH1-169 F	CGGCCAACAAGAAGATGAGT
hASH1-169 R	GCCATGGAGTTCAAGTCGTT

hNEUROG3-86 F	CTAAGAGCGAGTTGGCACTGA
hNEUROG3-86 R	GAGGTTGTGCATTCGATTGCG
h β-Actin-109 F	TTTTGGCTATACCCTACTGGCA
h β-Actin-109 R	CTGCACAGTCGTCAGCATATC
hsa-miR-200c-3p F	GCGCTAATACTGCCGGGTAAT
U6-F	CTCGCTTCGGCAGCACA
U6-R	AACGCTTCACGAATTTGCGT
miR universal reverse primer	From Agilent miR cDNA synthesis kit

F: represents forward primer sequence, and R: represents reverse primer sequence. h represents human, has-miR represents microRNA from human origin.

Table S2. List of ChIP primers used in the study.

ChIP Primers for miR-200c-3p Promoter	Primer Sequences 5′to 3′
hTBX2-controlmiR200c3pChip-145-F	TGGAAGCTTGCCCTAGAGTC
hTBX2-controlmiR200c3pChip-145-R	TCCAAATCCACCACCTCATT
hTBX2-681miR200c3pChip-179-F	CCGCTTTTTGTACCTCTGGA
hTBX2-681miR200c3pChip-179-R	AGGTGTGTCCTCCTGCCATA
hTBX2-931-miR200c3pChip-183-F	GGTAGCGGTGGGTAACTTCA
hTBX2-931-miR200c3pChip-183-R	CCAGAGAGATCCCTGAGGTC

F: represents forward primer sequence, and R: represents reverse primer sequence.

Table S3. List of the antibodies used in the study.

No.	Antibody	Catalog Number	Source
1.	SOX2 (E-4)	sc-365823	Santa Cruz Biotechnology, Inc. (Dallas, TX, USA)
2.	SOX2 (D1C7J)	14962	Cell Signaling Technology (Danvers, MA, USA)
3.	N-MYC (D4B2Y)	51705	Cell Signaling Technology (Danvers, MA, USA)
4.	N-MYC (C-19)	sc-791	Santa Cruz Biotechnology, Inc. (Dallas, TX, USA)
5.	CD9 (D3H4P)	13403	Cell Signaling Technology (Danvers, MA, USA)
6.	CD81 (B-11)	sc-166029	Santa Cruz Biotechnology, Inc. (Dallas, TX, USA)
7.	ARF6 (D1266)	5740	Cell Signaling Technology(Danvers, MA, USA)
8.	THBS1 (D7E5F)	37879	Cell Signaling Technology (Danvers, MA, USA)
9.	Beta-Actin (13E5) HRP-Conjugate	5125	Cell Signaling Technology (Danvers, MA, USA)
10.	Antirabbit IgG HRP-linked	7074	Cell Signaling Technology (Danvers, MA, USA)
11.	Antimouse IgG HRP linked	7076	Cell Signaling Technology (Danvers, MA, USA)
12.	TBX2 (D3)	sc-514291	Santa Cruz Biotechnology, Inc. (Dallas, TX, USA)
13.	ZEB1 (D80D3)	3396	Cell Signaling Technology (Danvers, MA, USA)
14.	Mouse IgG	I5381	Millipore-Sigma (St. Louis, MO, USA)
15.	Rabbit IgG	I5006	Millipore-Sigma (St. Louis, MO, USA)
16.	N-MYC	NB200-109	Novus Biologicals, Centennial, CO, USA
